# Clinical Trials Registration

**DOI:** 10.1371/journal.pmed.0030157

**Published:** 2006-03-28

**Authors:** Frank W Rockhold, Ronald L Krall

**Affiliations:** **1**GlaxoSmithKlineKing of Prussia, PennsylvaniaUnited States of America

We read with great interest the recent Policy Forum by Karmela Krleža-Jerić in *PLoS Medicine* on clinical trial registration [ 1]. GlaxoSmithKline (GSK) is committed to enhancing the transparency of clinical trial information through protocol registration and through registration of the results of clinical trials.

We are, therefore, disappointed that the article suggests the pharmaceutical industry is reluctant to embrace greater transparency and disclosure. In September 2004, GSK became the first company, and we believe the first trial sponsor, to establish a publicly available, Internet-based clinical trial register to provide results from all GSK-sponsored clinical trials of marketed medicines. The register currently has over 2,000 records, and there are now similar registers and databases across industry. Indeed, as Krleža-Jerić notes, in January 2005, industry made a commitment to disclose clinical trial information (summary protocol and results) on clinical trial registers and databases, which has been followed by a further position paper in September 2005. This latter position paper confirms industry's support for the agreement reached at the World Health Organization (WHO) Technical Consultation Meeting on Clinical Trials Registration Standards held on 25–27 April 2005. Moreover, industry has launched a clinical trial portal to enable and to facilitate access to clinical trial information.

The article focuses on the registration of clinical trial protocol information, and we agree that it is important to alert physicians and patients of the opportunity to participate in and to serve as a public record to ensure results are publicly disclosed. However, we are surprised that Krleža-Jerić makes little mention of results registration. It is registration of results (not summary protocol information) that helps to ensure that researchers, physicians, and others are aware of all the relevant information from clinical trials of medical interventions and can review the literature appropriately—it is this which can affect patient care.

With regard to registration of summary protocol information, we can appreciate that the discussion and debate around the WHO minimal dataset and the five data elements may have given the impression that industry is not embracing the concept of transparency and disclosure. However, industry has made the point about competitively sensitive information for a very good reason—that disclosure of one or more of these five data elements early in the process of drug development may undermine medicine development. The discovery and development of medicines is fundamental in the social contract that the pharmaceutical industry has with society, and undermining this contract is not in the interests of patients and society in general. Nonetheless, it is important to recognise that delay of one or more data elements will be by exception only. Industry will, whenever possible, disclose all 20 data elements in the WHO minimal dataset. GSK, for example, will only delay registration of one data element for some early phase (exploratory) trials, and that has to be approved by our Chief Medical Officer, Ronald Krall. All GSK-sponsored trials that, to use the International Committee of Medical Journal Editors (ICMJE) terminology, are “clinically directive” will be registered with all 20 data elements. The GSK approach is fully aligned with the ICMJE policy. Therefore, we do not believe that an escrow mechanism or another elaborate mechanism is required or justified for such a small number of data elements in such a small number of trials. We would, however, be concerned if in practice the frequency of delays was high. Therefore, a pragmatic way forward would be to review practice after 12–18 months to assess the extent of the issue (if any).

We hope these comments are helpful and constructive. We would be happy to discuss our views with the author in greater detail at any time if that would be helpful.
